# Changes in postgraduate medical education and training in clinical radiology

**DOI:** 10.2349/biij.4.1.e19

**Published:** 2008-01-01

**Authors:** D Lindsell

**Affiliations:** Faculty of Clinical Radiology, Royal College of Radiologists, London, United Kingdom

**Keywords:** Postgraduate, radiology, training, education

## Abstract

Postgraduate medical education and training in many specialties, including Clinical Radiology, is undergoing major changes. In part this is to ensure that shorter training periods maximise the learning opportunities but it is also to bring medical education in line with broader educational theory. Learning outcomes need to be defined so that there is no doubt what knowledge, skills, attitudes and behaviours are expected of those in training. Curricula should be developed into competency or outcome based models and should state the aims, objectives, content, outcomes and processes of a training programme. They should include a description of the methods of learning, teaching, feedback and supervision. Assessment systems must be matched to the curriculum and must be fair, reliable and valid. Workplace based assessments including the use of multisource feedback need to be developed and validated for use during radiology training. These should be used in a formative and developmental way, although the overall results from a series of such assessments can be used in a more summative way to determine progress to the next phase of training. Formal standard setting processes need to be established for ‘high stakes’ summative assessments such as examinations. In addition the unique skills required of a radiologist in terms of image interpretation, pattern recognition, deduction and diagnosis need to be evaluated in robust, reliable and valid ways. Through a combination of these methods we can be assured that decisions about trainees’ progression through training is fair and standardised and that we are protecting patients by establishing national standards for training, curricula and assessment methods.

## INTRODUCTION

Postgraduate medical education and training is undergoing major changes in many countries around the world. The old model of learning through an apprentice-ship relationship, with one or more senior clinical colleagues over very long working hours and seeing large numbers of normal and pathological cases, is being challenged. With limits on the hours that can be worked and shortened training as much time as possible at work must be used for learning. There also needs to be an appropriate assessment system to evaluate this learning.

A seamless process is required to take students through their basic undergraduate medical training into their early general postgraduate training and then on to specialist training or training for general practice. The process aims to produce fully trained doctors who can improve the healthcare of the population that they serve. The process does not stop there but continues with maintenance of those skills and development of new skills. This process should be demonstrable to the public.

## BASIC PRINCIPLES

There is a wealth of educational theory about how best to deliver training and how to assess the training outcome but this is less well developed when applied to medical education than in other spheres of education, although this situation is changing now.

The principles of good medical education and training encompass many different elements. Selection processes at whatever level, where there is open competition, need to be valid, open, objective and fair. Clear learning outcomes should be outlined so that there is no doubt as to what knowledge, skills, attitudes and behaviours are expected of those entering training.

The assessment systems must closely match the curriculum and should be fair, reliable and valid. The curricula should reflect the skills, knowledge, care and behaviour expected of doctors. Those who deliver teaching and training should have the appropriate skills and attitudes and standards should be determined for these skills. All of these elements should be regularly assessed and quality assured to ensure that they meet the pre-determined standards for each component of medical education.

In addition, medical education and training should reflect the diversity of the society in which the doctor is practising. This includes patient-focused care, learner-focused learning and making access to education and training as well as clinical care equally available to those from different parts of that society. There should also be equal opportunities for those with disabilities.

## THE CURRICULUM

The syllabus and curriculum need to be distinguished. A syllabus is simply a list of topics to be studied. Much has been written in the literature about the different types of curriculum [1] but in practice, the curriculum states the aims, objectives, content, outcomes and processes of a training programme. It includes a description of the methods of learning, teaching, feedback and supervision. It should describe the knowledge, skills, attitudes and behaviours that the learner will achieve.

In the United Kingdom, curricula are based on the General Medical Council’s ‘Good Medical Practice’ criteria [2] as well as the subject matter of the individual specialty. These criteria include good clinical care, maintaining good medical practice, relationships with patients, working with colleagues, teaching and training, being honest, sincere and having strong moral principles and being in good health.

Shortened working hours mean that training needs to be more formally structured to ensure full coverage of the curriculum in a shorter time period. Previously long hours allowed exposure to many different clinical conditions almost irrespective of the formal training but at the expense of fatigue, which has potentially detrimental effect on patient safety as well as learning capability.

Curricula should ideally be developed into competency or outcome-based models which can include generic elements related to ‘professionalism’ and other specialty-specific educational components.

In the United Kingdom, the Postgraduate Medical Education and Training Board (PMETB) has defined eight standards for curricula [3] i.e., the rationale, the learning content, the model of learning, the learning experiences, supervision and feedback, the management of curriculum implementation, the process of curriculum review, and update and conformity of the curriculum with equality and diversity legislation.

All curricula must demonstrate compliance with these standards before they can be approved by the PMETB. The rationale for the curriculum should explain the purpose of the curriculum, how it was developed and the appropriateness to the stage of learning and the particular specialty. It must set out the general professional and specialty-specific content to be covered, the intended learning outcomes and recommended learning experiences. There must be mechanisms for ensuring appropriate supervision of and feedback on learning to individual trainees. There should be regular curriculum review and revision where appropriate.

At the present time, most countries have a core radiology curriculum covering the breadth of general radiological experience supplemented by sub-specialty curricula based primarily on body systems. This model fits best with the radiologist becoming an equal member of a multidisciplinary team. As more and more clinicians acquire diagnostic imaging interpretative skills, radiologists need to ensure that their skills are better in order to justify their inclusion in the team. At the same time, ‘super’ specialisation runs the risk of “de-skilling” in non-specialist areas. For this reason, the challenge of a curriculum is to deliver the knowledge, skills and attitudes not only appropriate to the specialist area but also to ensure competence in the core areas of emergency radiology. This is particularly important wherever radiologists are in short supply to ensure that all sub-specialty areas are continuously covered.

There needs to be an assessment system, which is matched directly to the curriculum, that not only acts as a developmental tool for those in training but also as an assurance of competence in intended areas of clinical practice.

## THE ASSESSMENT METHODS

It is in the area of assessment that trainees and trainers will notice the greatest differences in the future. The apprenticeship method of learning, with a number of exams during the course of training to act as a stimulus to the acquisition of knowledge as well as hurdles to be crossed at variable times during training, is not sufficient on its own in an era of shortened training periods and greater public accountability.

Continual assessment, both formative and summative, is now the norm with the objective of ensuring clinical competence. Much of the terminology used by medical educationalists is new to many doctors and at times threatening because it is poorly understood. Schuwirth [4] uses the useful analogy of seeing assessment as a measurement of medical competence and then regarding examinations as the diagnostic tools for ‘medical incompetence’. As with all diagnostics, examinations have false positive and false negative results with the result that some competent trainees fail while some incompetent ones pass. These errors need to be minimised as much as possible as their consequences are serious. One way of doing this is to calculate the reliability and evaluate the validity of an examination or other assessment process.

### Reliability and Validity

High reliability of an assessment process means that it would reach the same conclusion if it were possible to administer the same test again to the same individual in the same circumstances or at the very least that the ranking of best to worst scoring students would not change. The assessment must be reproducible. Reliability is expressed as a co-efficient varying between 0 (no reliability) to 1.0 (perfect reliability).

Many assessments will state their ‘Cronbach alpha’ coefficient as an indicator of their reliability [5]. An appropriate cut-off for high stakes assessments is usually taken as greater than 0.8. One factor to improve reliability is to increase the testing time to ensure wide content sampling and sufficient individual assessments by different assessors. It has been shown that the reliability of multiple choice questions (MCQs) increases from 0.62 after one hour of testing to 0.93 after 4 hours while that of an oral exam, from 0.50 after one hour to 0.82 after 4 hours. Immediately, it can be seen that we now work in an era of the psychometrician and statistician guiding us in developing robust assessments.

Validity on the other hand, is a conceptual term which cannot be measured but is an indicator of whether the assessment tests what it is meant to test. A number of different facets of validity has been described [6] implying that multiple sources of evidence are required to evaluate the validity of an assessment.

As well as evaluating the reliability and validity of an assessment system, the educational impact, cost efficiency, acceptability and feasibility should also be evaluated. Optimising an assessment method is about balancing these six components. High stakes pass or fail examinations need high reliability and validity whereas a formative developmental assessment relying more on feedback to a trainee can focus more on educational impact and less on reliability.

### Standard Setting

Formal standard setting processes need to be developed for summative assessments. Standard setting is the process used to establish the level of performance required by an examining body for an individual trainee to be judged as competent. It is in effect the pass mark. Many methods of standard setting have been described [7]. Relative standards are based on a comparison among the trainees taking that examination, and they pass or fail in accordance with how they perform in relation to each other. For example, the top 80% always pass the examination.

The preferred standard is an absolute one where trainees pass or fail according to their own individual performance irrespective of how others perform. For this, a formal standard setting process is required and probably the best known is the Angoff method [9]. Assessors are asked to make judgements, as subject experts, as to the probability of a ‘just passing’ trainee answering a particular question or performing the indicated task correctly. The assessors’ mean scores are used to calculate a standard for the question.

### Assessment Methods

**Figure 1:**
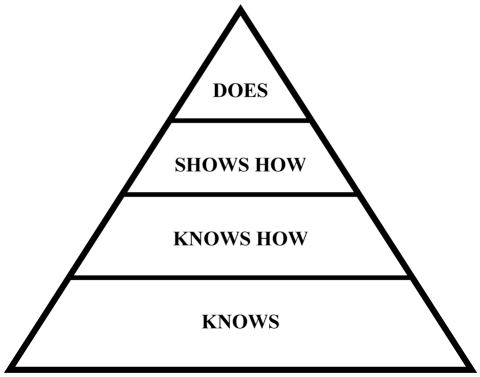
Miller's Pyramid.

In determining the form of assessment to use, it must be decided whether knowledge, competence or performance is being assessed. Miller’s Pyramid [8] (Figure 1) is a useful way of describing levels of competence. This describes the progress from ‘Knows’, which reflects applied knowledge through to ‘Knows How’, which requires more than just knowledge to ‘Shows How’, which requires an ability to demonstrate a clinical competency through to ‘Does’.

Each stage requires a different form of assessment and, ultimately, a test of clinical performance at the ‘Does’ stage, which reflects what the doctor actually does in the workplace. There is much debate about the format of question that is the best in testing medical competence but, in fact, if the content of the items is similar, it has been shown that the question format is almost completely unimportant [10]. In practice, MCQs tend to be used to test factual knowledge with workplace observations of practice being used in a formative way to feedback to the trainee their developmental needs. In parallel, an assessment of behaviours and attitudes is best undertaken through multi-source feedback. Presentations and oral exams also have their place but their relatively low reliability means that high stakes decisions should not be based solely on their results.

Workplace-based assessment methods have been used in a number of medical specialties. The mini-Clinical Evaluation Exercise (mini-CEX) was developed in the United States to assess the clinical skills that trainees most often use in real clinical encounters. Trainees are observed directly by an assessor when they are undertaking tasks such as history taking, clinical examination and communicating with patients. Each encounter takes 15 to 20 minutes and should be repeated on a number of occasions in different clinical situations with different assessors.

**Figure 2:**
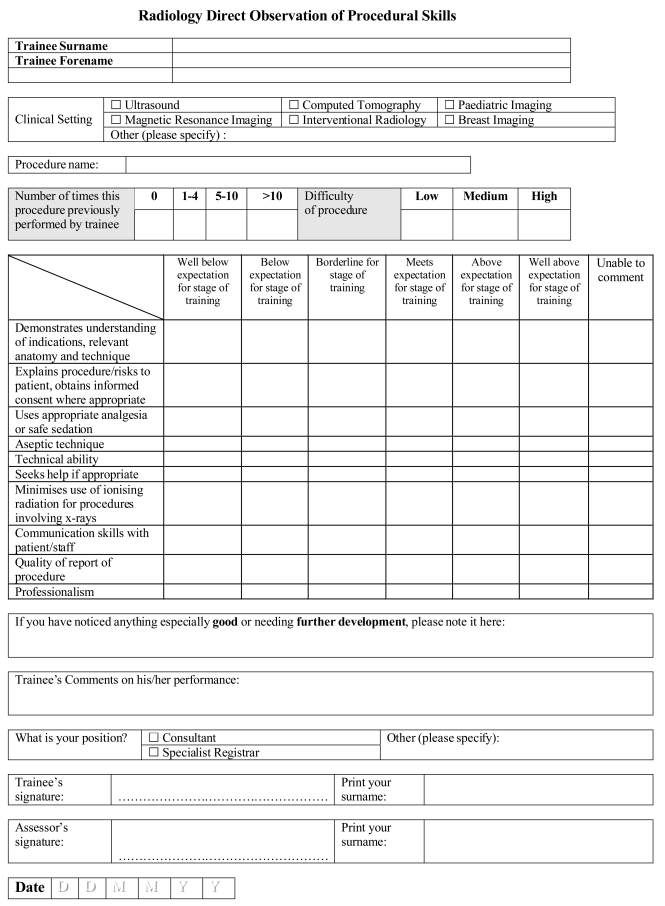
Radiology Direct Observation of Procedural Skills.

The mini-CEX has been shown to have good reproducibility, validity and reliability in general medicine. It has been shown that for a given area of performance at least four assessments are needed if the trainee is doing well and more than four if the trainee is falling below the required standard. Directly Observed Procedural Skills (DOPS), which has been developed by the Royal College of Physicians in the United Kingdom, requires an assessor to observe directly a trainee undertaking a procedure and then grade the performance of specific pre-determined components of the procedure. These include generic skills such as consent and communication as well as the practical aspects of the procedure itself. An example to be piloted in the United Kingdom is appended (Figure 2).

In a Case-Based Discussion (CBD) a selection of the trainee’s cases are discussed in a standardised and structured oral assessment. A trained assessor questions the trainee about the care provided in pre-defined areas – problem definition (diagnosis), clinical thinking (interpretation of findings), management and anticipatory care (treatment and care plans).

Multi-source feedback (MSF) is an objective systematic collection and feedback about an individual’s performance derived from a number of people (‘raters’) working with individuals from a variety of different backgrounds e.g., clinical colleagues, nurses, radiographers and clerical staff. This method permits an assessment of generic skills such as communication, leadership, team work, teaching, punctuality and reliability.

The responses from about 15 ‘raters’ are required to ensure a reliable assessment of the individual’s attitudes and behaviours. The raters are asked whether they have no concerns, some concerns or major concerns about the individual in areas such as showing respect for patients’ opinions, privacy, dignity and confidentiality, giving appropriate and understandable information to patients, respecting other team members’ roles and working well as part of a team, and being readily available and accepting of responsibility for his/her actions. Some MSF will also assess specialty-specific attitudes and behaviours. Other possible workplace-based assessment methods include audit, presentation and teaching assessments, and patient satisfaction questionnaires.

Although workplace-based assessments are primarily formative developmental processes, the accumulated knowledge of a trainee's performance in these assessments can feed into the formal summative assessments that determine the progress of a trainee from one stage of training to another, and ultimately as being ‘licensed’ for independent practice through whatever process exists in the host country for certification. Such decisions require information from a variety of different sources, the so-called triangulation of evidence, to be robust. This means that information from workplace-based assessments, multi-source feedback, examination results, evaluation of audit and teaching skills, outcomes data, patient questionnaires and reports from educational supervisors, or at least some of these, are required.

## WHAT DOES THIS ALL MEAN FOR RADIOLOGY?

The questions for radiology are how much of this applies to the specialty and what adaptations need to be undertaken to suit the uniqueness of the specialty?

In many ways radiology is different to other specialties. Trainees are protected in their early years by working in a close apprenticeship relationship with their trainers and their knowledge and skills in the workplace are being assessed on a daily basis by their trainer but this may not be done in a standardised way and it may not be formally documented.

Radiologists require different skills such as perceptual and observational skills and the ability to recognise patterns or abnormalities. Having made such observations, they need to make appropriate deductions from those observations and from the clinical information available to them to reach a differential and possibly a definitive diagnosis, and finally they need to make appropriate recommendations for further investigation or management. In addition, they require practical skills to undertake diagnostic and interventional procedures.

Many of the diagnostic skills are assessed on an on-going basis during training through the use of ‘film viewing tests’ and other interactions with their trainer. The weakness of such assessments is that they are usually locally derived and there are usually no national standards. There is a considerable amount of radiology teaching material available through e-learning resources, and on DVDs and CDs.

In the United Kingdom, the curriculum for the first three years is available to UK trainees through an electronic learning database, a joint project between the Department of Health and the Royal College of Radiologists. This permits learning pathways to be developed and also a degree of self assessment, which can be recorded through a learning management system. It is hoped that the next phase of this project will be to complete a large archive of validated cases, which should permit the standardisation of assessments for both trainees and trained specialists, who may need to demonstrate on-going competence.

In the interim, those responsible for radiology curricula should define the core diagnostic skills in each area of the curricula for each stage of training on which assessments can be based. Workplace-based assessments such as Directly Observed Procedural Skills (DOPS) lend themselves ideally to the assessment of diagnostic and interventional radiological procedures and with some adaptation so do mini-CEX and CBD. MSF and the assessment of audit and teaching skills are generic to all specialties. The knowledge base, which underpins a competent radiologist, is vast and MCQs in the format of single best answer appear to be the most reliable and valid way of assessing this.

The film viewing components of any radiology examination need to move to a digital format ideally allowing image manipulation, where appropriate, in order to simulate the workplace as closely as possible. An electronic examination should allow more candidates to be examined on the same material to ensure better standardisation. Oral examinations suffer from having poor reliability and are disappearing from the assessment processes of many medical specialties.

It can be argued that in radiology oral examinations are being used to assess something unique that cannot be assessed in any other way. They are assessing the day-to-day interactions that take place between a radiologist and a clinician where the radiologist interprets an imaging test on the basis of a certain amount of clinical information and from that may reach a diagnosis or may need to obtain more clinical information from the clinician to do this or to recommend further investigation.

The oral exam allows simulation of this interaction and allows the examiner to assess the level of confidence that a candidate has in reaching a diagnosis. The challenge is to ensure that as many candidates are examined on the same material as is possible, and that they are examined over as broad a spectrum of the curriculum as is possible to ensure as high a reliability as is achievable with this form of assessment.

Radiology is in a unique position to combine what was best in the past, in terms of the close mentoring and apprenticeship of trainees, with what is the best of the new methods in terms of workplace-based assessments, examinations and multi-source feedback. Combining these various assessments assures us that decisions about trainees’ progression through training is fair and standardised, and that we are protecting patients by establishing national standards for training, curricula and assessment methods.
